# Predicting Transitions in Low and High Levels of Risk Behavior from Early to Middle Adolescence: The TRAILS Study

**DOI:** 10.1007/s10802-012-9624-9

**Published:** 2012-03-17

**Authors:** K. Monshouwer, Z. Harakeh, P. Lugtig, A. Huizink, H. E. Creemers, S. A. Reijneveld, A. F. De Winter, F. Van Oort, J. Ormel, W. A. M. Vollebergh

**Affiliations:** 1 Interdisciplinary Social Science, Utrecht University, PO Box 80.140, 3508 TC Utrecht, The Netherlands; 2 Trimbos Institute (Netherlands Institute of Mental Health and Addiction), Utrecht, The Netherlands; 3 Department of Methods and Statistics, Utrecht University, Utrecht, The Netherlands; 4 Department of Child and Adolescent Psychiatry, Erasmus Medical Centre, Rotterdam, The Netherlands; 5 Research Institute of Child Development and Education, University of Amsterdam, Amsterdam, The Netherlands; 6 Department of Health Sciences, University Medical Center Groningen, University of Groningen, Groningen, The Netherlands; 7 Interdisciplinary Center for Psychiatric Epidemiology, Department of Psychiatry, University Medical Center Groningen, University of Groningen, Groningen, The Netherlands

**Keywords:** Multiple risk behavior, Adolescence, Predictors, Latent transition analysis, Longitudinal data

## Abstract

The present study examined the joint development of substance use and externalizing problems in early and middle adolescence. First, it was tested whether the relevant groups found in previous studies i.e., those with an early onset, a late onset, and no onset or low levels of risk behavior could be identified, while using a developmental model of a single, underlying construct of risk behavior. Second, departing from Moffitt’s taxonomy of antisocial behavior, it was tested if early, but not late, onset risk behavior is predicted by a problematic risk profile in childhood. Data were used from TRAILS, a population based cohort study, starting at age 11 with two follow-ups at mean ages of 13.6 and 16.3 years. Latent transition analyses demonstrated that, both in early and middle adolescence, a single underlying construct of risk behavior, consisting of two classes (labeled as low and high risk behavior), adequately represented the data. Respondents could be clearly classified into four possible transition patterns from early to middle adolescence, with a transition from high to low being almost non-existent (2.5 %), low to low (39.4 %) and low to high (41.8 %) being the most prevalent, and high to high (16.2 %) substantial. As hypothesized, only the high-high group was characterized by a clear adverse predictor profile in late childhood, while the low-high group was not. This study demonstrates that the development of substance use is correlated with externalizing problems and underscores the theory that etiologies of early and later onset risk behavior are different.

## Introduction

Experimenting with substance use and other types of risk behavior are common in adolescence (Hibell et al. 2009). However, longitudinal studies have demonstrated that there are marked individual differences in the developmental patterns of these behaviors. In their taxonomy of antisocial behavior, Moffitt (1993) and Moffitt et al. (2002) demonstrated that two prototypes of offenders, the life-course-persistent (LCP: childhood onset and continuing into adulthood) and adolescent-limited group (AL: beginning in adolescence and desisting in young adulthood), account for the bulk of antisocial behavior (two additional groups were identified and labeled abstainers and low-level chronics, but these groups were relatively small, i.e., around 5 % and 8 % respectively). The life-course persistent antisocial group was characterized by high-risk childhood backgrounds (e.g., inadequate parenting, neurocognitive problems, and temperament and behavior problems) and appeared to be at highest risk for adverse outcomes in adulthood, whereas the adolescent onset group was considered to be quite normative, and thus at much lower risk for such adverse outcomes (Moffitt and Caspi 2001; Moffitt et al. 2002; Odgers et al. 2008). While Moffitt’s taxonomy focuses on antisocial behavior, a number of studies have demonstrated the usefulness of this taxonomy for developmental patterns of substance use. Accordingly, Wanner et al. (2006), in a study on alcohol and marijuana use and gambling, identified three trajectories: early initiation (around age 11), late onset (around age 14), and consistently low involvement. Flory et al.(2004) also identified three subgroups for both alcohol and marijuana: early onset, late onset and non-users. In addition, clear differences between the predictor profiles confirmed that, compared to the abstainers and late onset groups, the early onset substance use group appeared to be at much higher risk for adverse childhood predictors (revealing a problematic profile), including lower levels of parental knowledge about adolescents’ activities and self-esteem and higher levels of novelty seeking and conduct disorder (Flory et al. 2004; Wanner et al. 2006).

Empirical studies in the adolescent population have established that different types of risk behavior cluster, i.e., those involved in one type of risk behavior often show other types of risk behavior as well (e.g., Donovan et al. 1998; Lynskey et al. 1998; Van Nieuwenhuijzen et al. 2009; Vazsonyi et al. 2008). One of the hypotheses for the interrelationships among various types of risk behavior is that they have common causes or correlated risk factors (Jessor and Jessor 1977; Palmer et al. 2009). Very few studies on co-occurring risk behaviors were based on longitudinal data, and the majority of those that did focused on substance use (Duncan et al. 1998; Palmer et al. 2009; Wanner et al. 2006). Wanner et al. (2006) found evidence for joint trajectories of gambling, alcohol and marijuana use. Duncan et al. (1998) demonstrated that a single substance use behavior factor (indicated by alcohol, cigarette and marijuana use) adequately represented change in the use of these substances during adolescence. Palmer et al. (2009) showed that adolescents and young adults are not specialized users, but rather tend to use or abuse multiple substances increasingly with age. Involvement with multiple substances appeared to be driven by common or correlated risk factors (Palmer et al. 2009). There are very few developmental studies based on a broader risk behavior concept, i.e., including not only substance use but also other types of adolescent risk behavior. In fact, we found only one study investigating the joint development of substance use and antisocial behavior (i.e., conduct problems) in adolescence (Wu et al. 2010). The authors found that the majority of students showed parallel trajectories of conduct problems and substance use (i.e., stable, high; parallel increase; and stable, low), while a fourth group showed minimal conduct problems and increasing substance use (Wu et al. 2010). Higher levels of childhood conduct problems during kindergarten predicted a greater probability of expected membership in the most problematic class (high conduct and high substance use problems) relative to the less problematic classes (Wu et al. 2010).

In conclusion, it has been clearly established that substance use and antisocial behavior often co-occur in adolescents. However, developmental studies have focused almost solely on trajectories of either substance use or antisocial behavior and not on the commonality of the two types of risk behavior. This gap in the literature warrants further investigation as correlated change in both would underline the need to take the co-occurrence of antisocial behavior into account when explaining the adverse consequences of substance use, and vice versa. Furthermore, it is important to identify the characteristics of those who show adverse trajectories across several risk behaviors, and in particular of those who do so at an early age, as this group appears to be particularly at risk for adverse outcomes later in life.

The objective of the present study was twofold: first, using latent transition analysis (Lanza et al. 2009), we tested whether the relevant groups found in previous studies (e.g., Flory et al. 2004; Moffitt et al. 2002), i.e., those with an early onset, a late onset, and no onset or low levels of risk behavior could be identified, while using a developmental model of a single, underlying construct (constituted by tobacco, alcohol and marijuana use and externalizing behavior problems). If so, in a second step, we tested the hypothesis that those with an early onset of risk behavior (i.e., in early adolescence), persisting into middle adolescence, were characterized by a problematic risk profile in late childhood, while those with a later onset, i.e., in middle adolescence were not. Previous research has suggested that parental and child functioning factors are the most significant predictors of early onset risk behavior (i.e., prior to adolescence), as opposed to more distal factors such as the neighborhood environment and social status among peers (Kaplow et al. 2002). Therefore, we assessed associations with factors from the family domain (i.e., parenting practices, parental substance use), the individual domain (e.g., personality and childhood behavior), as well as demographic factors (i.e., gender, age, socio-economic status and ethnic background). All research questions were addressed by analyzing data from a large (*n* = 2,230), population based cohort study of Dutch adolescents, starting at age 11 with two follow-ups at the mean ages of 13.6 and 16.3 years, thereby capturing the majority of onset and continuation of adolescent risk behaviors.

## Methods

### Study Design and Population

The present study is based on the data of the first, second and third waves of the TRacking Adolescents’ Individual Lives Survey (TRAILS). Data collection for the first assessment started in 2001. The second and third assessment waves took place at intervals of about 2.5 years. Informed consent was obtained from all participants at each assessment wave. The Central Dutch Medical Ethics Committee has approved the study.

The target sample comprised young adolescents from five municipalities in the north of the Netherlands, including both urban and rural areas. The sample selection involved two steps. First, the municipalities were requested to provide names and addresses of all inhabitants born between 1 October 1989 and 30 September 1990 (first two municipalities) or between 1 October 1990 and 30 September 1991 (last three municipalities), which yielded 3,483 names. Simultaneously, primary schools within these municipalities were approached with a request to participate. Of the 135 schools, 122 (90.4 %) agreed to participate, accounting for 90.3 % of the adolescents. Of all subjects who were approached (*n* = 3,145), 6.7 % were excluded because of incapability or language problems. Of the remaining 2,935 children, 2,230 (76.0 %) children and their parents agreed to participate, and all were included in the study (mean age 11.1 years, *SD* = 0.6, 50.8 % female). Responders and non-responders did not differ with respect to the prevalence of teacher-rated problem behavior or associations between socio-demographic variables and mental health indicators. A high response rate (96 %) was yielded at the second assessment (*n* = 2,149, mean age 13.6 years, *SD* = 0.5, 51.2 % female). For the third assessment (*n* = 1,816, mean age 16.3 years, *SD* = 0.7, 52.3 % female), the response rate was somewhat lower (81.4 %). Further details on the sample and fieldwork procedure have been published elsewhere (De Winter et al. 2005).

### Procedure

At the first assessment, trained interviewers visited one of the parents or guardians in their homes to conduct an interview. In addition, the parent completed a written questionnaire. At the second and third assessment parents were asked to fill out a questionnaire, which they received by mail. At each of the three assessments, the self-report questionnaires were completed by the adolescents at school, in group settings under the supervision of a TRAILS assistant.

### Measures

#### Risk Behavior

The putative indicators of risk behavior at wave 2 and 3 included self-report measures on tobacco, alcohol and cannabis use, and externalizing behavioral problems. *Alcohol use* was assessed by the question ‘How often did you drink alcohol in the last 4 weeks? (14 categories, ranging from 0 to 40 times or more) (O’Malley et al. 1983). *Tobacco use* was measured by the question ‘How many cigarettes did you smoke on average in the last 4 weeks (7 categories: never smoked, did not smoke in the last 4 weeks, less than 1 cigarette a day, 1–5, 6–10, 11–20, more than 20 cigarettes a day). *Cannabis use* was assessed by the question ‘How many times in the past year have you used weed or hashish? (14 categories, ranging from 0 to 40 times or more) (O’Malley et al. 1983). *Externalizing behavioral problems* were assessed in the Youth Self Report (YSR; Achenbach 1991). The concept of externalizing behavioral problems refers to a grouping of behavior problems that are manifested in children’s outward behavior and reflect the child negatively acting on the external environment (Liu 2004). Other terms used to describe this behavior include conduct problems, antisocial and undercontrolled (Hinshaw 1987). The YSR has shown good reliability and validity (Achenbach 1991), also in the Dutch context (Verhulst et al. 1997). The YSR contains 101 problem items (3 categories: not true (scored as 0), somewhat true (scored as 1), very true or often true (scored as 2), on the basis of the preceding 6 months), of which 32 items measure externalizing behavioral problems. The externalizing broad band scale consists of the scales delinquent behavior (e.g., ‘I steal from home’) and aggressive behavior (e.g., ‘I fight a lot’). The delinquent behavior scale contains three items on substance use. To avoid spurious associations, these items were omitted. The final score was calculated as the average of the scores on the remaining 29 items, thus the respondents’ final score on the scale could range from 0 to 2 (alpha = 0.85 at T2 and alpha = 0.86 at T3).

### Predictor Variables

The putative predictor variables were measured at baseline (T1) and were based on self-reporting and reports by one of the parents (96 % mothers). Individual factors included: age, gender coded as 1 (girls) and 0 (boys), aggressive behavior at age 4–5 years (4 item scale, alpha = 0.70), assessed by asking the parents whether their child was short-tempered, obedient, bullying other children or overbearing at the age of 4–5 years. Substance use at T1 was based on self-report and included alcohol use (at least one glass), tobacco use and marihuana or other drug use, coded as 0 (never used) and 1 (used at least once). Externalizing behavioral problems at T1 were assessed by the YSR (32 items, alpha = 0.85) (Achenbach 1991). Temperament was assessed by the parent version of the short form of the Early Adolescent Temperament Questionnaire-Revised (EATQ-R; Putnam et al. 2001). Of interest for the present study were the dimensions (1) high-intensity pleasure, defined as the pleasure derived from activities involving high intensity or novelty (6 items, alpha = 0.77), (2) shyness, which refers to behavioral inhibition to novelty and challenge, especially social (4 items, alpha = 0.84), (3) effortful control, defined as the capacity to voluntarily regulate behavior and attention (11 items, alpha = 0.86) (Creemers et al. 2009). Parent reported family factors included socio-economic status (SES), which was based on the parent report and calculated as the mean of income, educational, and occupational level of each parent at wave 1 (Ganzeboom and Treiman 1996), and was categorized into low (1), average (2), and high (3). Ethnic minority was based on the country of birth of the father and the mother. Those who had one or both parents born in a non-Western country were classified as belonging to an ethnic minority group (coded as 1); all other respondents were coded as 0. Parents divorced was assessed by inquiring about the marital status of the reporting parent and coded as 1 (divorced) and 0 (not divorced). The category ‘divorced’ also included biological parents who were never married, had lived together as a couple, but were separated. Perceived parenting behaviors were based on children’s report and assessed with three scales of the children version of the Egna Minnen Beträffande Uppfostran (EMBU-C). Good reliability and validity of this instrument (Castro et al. 1993) were confirmed for the Dutch translation (Markus et al. 2003). The scale of emotional *warmth* contained 18 items (alpha = 0.91 for each of the parents). Sample items include ‘Do you think your parents love you?’; ‘If your parents punish you, are they always fair?’ The scale of *emotional rejection* contains 17 items (alpha = 0.84 for each of the parents). Sample items include ‘Do your parents punish you for minor things?’; ‘If something goes wrong at home, are you usually the one who gets blamed for it?’ The scale of *overprotection* contains 12 items (alpha = 0.70 for the father and alpha = 0.71 for the mother). Sample items include ‘Are your parents very concerned about your physical health?’; ‘If your parents are sad, do you sometimes think it’s your fault?’ Because of the high associations (warmth: *r* = 0.79; rejection *r* = 0.68; protection *r* =0.80, *p* < 0.01), the scores of the father and mother were combined into a single measure. *Maternal/paternal smoking* was coded as 0 (not smoked during the last year) and 1 (smoked during the last year). *Maternal/paternal alcohol use* was coded as 0 (no alcohol use during the last year) and 1 (drank alcohol during the last year).

### Analyses

Because of missing data in both the predictors and indicators of risk behavior, we used multiple imputed data. Data were imputed under the saturated model using the Bayesian module of Mplus 6 (Muthén and Muthen 2010a, b). All further analyses used the 5 imputed data sets, and parameter estimates were pooled over these datasets (Muthén and Muthén 2010a, b). The Maximum Likelihood Robust (MLR) estimator was used to deal with skewness and kurtosis in the data.

In order to assess whether meaningful latent risk behavior statuses could be identified, both at T2 and T3, and to study how individuals change latent status membership over time, we applied Latent Transition Analysis (LTA) as implemented in Mplus version 6.1 (Muthén and Muthén 2010a, b).

LTA is a longitudinal extension of latent class analysis, ideally suited for modeling multivariate behavior constructs developmentally (Collins and Lanza 2009; Lanza et al. 2009; Velicer et al. 1996). LTA is a relatively novel approach in the social sciences but has been increasingly applied in recent studies (e.g., Chung et al. 2005; Connel et al. 2008; Dishman et al. 2009; Goldweber et al. 2011; Händel et al. 2009; Lanza et al. 2009).

For the present study, we were interested in separating the respondents into two groups, based on their score on the four indicators 1) smoking tobacco (number of cigarettes) and 2) frequency of alcohol use over the last month, 3) last year frequency of cannabis use and 4) score on the externalizing problem behavior scale. Because of the high occurrence of ‘zeroes’ (i.e., no risk behavior) in the data of the three substance use indicators, the link function between the indicators and the latent variables ‘risk behavior’ was specified as a zero-inflated Poisson function. The loadings of these parameters were furthermore constrained across the two measurement occasions, so that the definition of the latent risk behavior factors is equivalent across time. Comparison between a model with indicators freely estimated at each time and a model where indicators were constrained to be equal at times indicated that such measurement invariance was supported by the data (unconstrained model: BIC 32907, Df = 65 versus constrained model: BIC 33037, Df = 59). We labeled the class with the lower scores on the risk indicators as the low level class and the class with the higher scores as the high level class. This resulted in four transition probability patterns from T2 to T3: low-low, low-high, high-low, and high-high. From a theoretical perspective we considered the two class option to be the most relevant. However, we also tested whether this choice was appropriate from a statistical point of view by comparing the statistical fit of the two class model (resulting in a low and high level class) to solutions with more and fewer classes. By default, the absolute fit of a model will improve when the number of classes increases. In fact the model where each case has its own class will show the best fit. Therefore, when evaluating these models, not the absolute fit, but differences between the models in terms of relative fit are most informative. We found that the three, four and five class solutions showed a slightly better fit (BIC values respectively 32405, 32362 and 32806, Df = 59) compared to the two class solution (BIC 33037, Df = 59), but the differences between these models in terms of fit were small. A closer inspection showed that, when using more than two classes, some transition classes contained very few respondents or were even empty. Based on these findings we decided that the two class model is our preferred model.

In order to analyze specific transition patterns, we post-processed the results of the LTA analysis for all five imputed datasets to SPSS version 18.0 (PASW Statistics 18—SPSS 2009). Based on their most likely class membership, individuals were classified according to the four T2-T3 transition classes. These transition classes were included as an outcome variable in a multinomial logistic regression analysis, where the five imputed datasets were pooled. The reference group was specified to be the low-low group, so each odds ratio can be interpreted as the effect of the covariate on the odds of membership in a particular transition class relative to membership in the low-low transition class. All predictor variables were entered simultaneously in the regression model.

## Results

### Sample Characteristics

At baseline (T1), 10.6 % were of non-Western origin; approximately a fifth (21.3 %) of the sample had parents who were divorced, and 25 % were living in a low socio-economic status family. At T1, almost one third of the sample (31 %) had used alcohol, 14 % had smoked tobacco and a very small percentage (1 %) reported having used cannabis or other drugs at least once in their lives. The mean score on the externalizing behavioral problem scale was 0.27 (*SD* = 0.19).

### Latent Risk Behavior Statuses and Transition Probabilities

At T2, almost a fifth of respondents (18.8 %) were classified in the high level risk behavior group. At T3, approximately two and a half years later, this percentage had almost tripled (59.6 %). Table 1 shows the sample means for all four transition possibilities. It is clear that a switch from low to high levels of risk behavior is associated with higher scores on each of the four indicators. There are large differences between these groups, indicating that the low and high level groups can be clearly distinguished. This finding is supported by the entropy value (0.74) (Nyland 2007). The entropy relates to the quality of the classification into different latent classes. A low value for the entropy (i.e. close to 0) implies that the classification is totally random, and that every respondent has an equal chance to be a member of one of the LTA classes. An entropy close to 1 means that everyone can be perfectly classified into one of the classes. The entropy that we find (0.74) implies that the classification is not perfect, but that most respondents can be clearly assigned to one of the classes. The largest group in the sample (41.8 %) comprises adolescents who made a transition from low level at T2 to high level risk behavior at T3. More than one third of the sample (39.5 %) had a low level status at both T2 and T3 and 16.2 % of the sample had a high level status at both T2 and T3. A very small percentage (2.5 %) made a transition from a high level status at T2 to a low level status at T3.

**Table 1:** Two-status model of risk behavior: sample means of the risk behavior indicators for all four T2 → T3 transition possibilities, weighted by most likely transition patterns

	Transition probability classes			
	Low → Low	Low → High	High → Low	High → High
Prevalence	39.5 %	41.8 %	2.5 %	16.2 %
Posterior sample means				
T2				
Tobacco use	0.12	0.24	2.00	2.67
Cannabis use	0.01	0.02	0.88	1.06
Alcohol use	0.48	0.75	3.67	3.74
Externalizing behavior	0.23	0.29	0.53	0.50
T3				
Tobacco use	0.18	2.30	0.25	3.42
Cannabis use	0.03	2.00	0.06	2.78
Alcohol use	1.21	5.05	0.99	4.86
Externalizing behavior	0.22	0.37	0.36	0.45

### Predictors of Transitions in Risk Behavior Status

Table 2 presents the results (odds ratios, adjusted for all other variables in the model) of the multinomial logistic regression analyses of most likely transition class membership based on the predictors. The results for the transition from high to low risk behavior class are not presented, as the size of this transition class was too small (2.5 %) to adequately perform the analyses. The odds ratios in Table 2 represent the effect of the predictor on the odds of transitioning to a particular class relative to membership of the low-low risk behavior class. Those with higher scores on effortful control were significantly less likely than those with lower scores to be in the high-high relative to the low-low class. On the other hand, those with divorced parents were significantly more likely than those with non-divorced parents, to be in the high-high relative to the low-low class. A particularly strong predictor of belonging to the high-high group was a higher score on externalizing behavioral problems at T1 (i.e., when respondents were around the age of 11 years). Furthermore, high-intensity pleasure, early onset of alcohol and tobacco use, maternal smoking and parental overprotection were predictive of belonging to the high-high class. Only one variable, i.e., high-intensity pleasure, was predictive of belonging to the low-high transition class.

**Table 2:** Predictors of transitions in risk behavior status from T2 → T3, multivariate model

T1 predictors	Transition classes (reference class is Low → Low)					
	Low → High			High → High		
	OR	95 % CI		OR	95 % CI	
Socio-demographic factors						
Female gender (p)	1.08	0.84	1.39	0.84	0.63	1.11
Higher age (p)	1.06	0.87	1.27	0.99	0.77	1.29
Higher socio-economic status (p)	0.93	0.78	1.11	0.83	0.64	1.06
Ethnic minority (p)	0.96	0.61	1.52	1.00	0.59	1.71
Individual factors (T1)						
Aggressive behavior at age 4/5 (p)	1.13	0.93	1.36	1.27	0.99	1.61
Shyness (p)	0.97	0.87	1.10	0.83	0.68	1.03
High-intensity pleasure (p)	1.18**	1.05	1.32	1.26*	1.06	1.51
Effortful control (p)	0.98	0.83	1.15	0.77*	0.62	0.95
Alcohol use (s)	1.47	1.10	1.97	1.96***	1.37	2.78
Tobacco use (s)	1.27	0.84	1.93	2.80***	1.81	4.32
Marihuana or other drug use (s)	1.46	0.38	5.58	1.15	0.30	4.48
Externalizing behavioral problems (s)	1.54	0.73	3.24	8.30***	3.35	20.56
Family factors (T1)						
Parents divorced (p)	1.12	0.79	1.58	1.82**	1.21	2.74
Maternal smoking (p)	1.18	0.91	1.54	1.66**	1.20	2.30
Paternal smoking (p)	1.12	0.86	1.46	1.32	0.90	1.94
Maternal alcohol use (p)	0.87	0.61	1.23	0.94	0.64	1.38
Paternal alcohol use (p)	1.20	0.67	2.17	1.29	0.62	2.68
Parental overprotection (s)	1.30	0.95	1.79	2.00**	1.28	3.13
Parental rejection (s)	0.89	0.58	1.36	0.60	0.33	1.09
Parental warmth (s)	1.00	0.75	1.33	0.87	0.60	1.24

*s* self report, *p* parent report

*p < 0.05, ** *p* < 0.01, *** *p* < 0.001

## Discussion

Our study revealed that, as expected, the development of substance use (smoking, drinking, cannabis use) and externalizing behavioral problems in adolescence can be adequately represented by a single, underlying construct of risk behavior. This finding indicates that the development of patterns of substance use is structurally related to the development of externalizing problems at this age. We found three substantial transition groups: consistently low levels of risk behavior (low-low), increasing levels of risk behavior (low-high) and consistently high levels of risk behavior (high-high). The fourth potential pattern, i.e., decreasing levels of risk behavior (high-low), appeared to be virtually absent. The high-high group was characterized by a clear predictor profile, while the low-high group was not. The latter finding supports the theory that early onset risk behavior, including substance use, is primarily explained by an underlying vulnerability towards risk behavior, while late onset risk behavior is rather normative (Moffitt 1993).

Externalizing behavioral problems in late childhood (i.e., around the age of 11 years) was by far the strongest predictor (odds ratio of 8.30) for membership of the high-high group. These childhood externalizing behavioral problems may express an underlying individual vulnerability toward risk behavior and thereby appear to be the most salient indicator for an increased risk of early onset risk behavior, including substance use. Moreover, the results of previous studies suggest that these childhood externalizing problems in themselves may also trigger early onset substance use (Fite et al. 2008). Our findings corroborate those reported by Wu et al. (2010), who also found that higher levels of childhood conduct problems predicted a greater probability of classification in the most problematic substance use and conduct disorder developmental class.

Aggressive behavior at the age of 4/5 years was not predictive of the high-high transition class. These findings may indicate that individual vulnerability develops and expresses itself in interaction with other risk or protective factors in the course of childhood, most likely in the home environment (e.g., poor parenting, parental divorce). Other studies have reported similar findings (Aguilar et al. 2000; Moffitt and Caspi 2001). Aguilar et al. (2000) found that differences between childhood onset and adolescent onset groups were not significant for neurocognitive and temperamental problems measured prior to the age of 3 years. Moffitt and Caspi (2001) found small effect sizes for ‘life-course persistent’ and ‘adolescent limited’ differences in behavioral risks in early childhood that increased with age. The authors suggest that discipline problems are incrementally exacerbated when children endure long-term adversity (Moffitt and Caspi 2001).

When compared with the low-low group, the high-high group was further characterized by a higher exposure to a number of risk factors including higher levels of high-intensity pleasure, lower levels of effortful control, alcohol and tobacco use, parental divorce, maternal smoking, and parental overprotection. In contrast, those making the transition from low to high involvement in risk behavior were only characterized by a somewhat higher level of high-intensity pleasure (odds ratios of 1.18). Thus, late onset risk behavior does not appear to be associated with a ‘risky’ personality profile or higher social or familial vulnerability. As suggested by Moffitt (1993), late onset risk behavior represents the wish of adolescents to appear mature, demonstrate autonomy from parents and other authority figures, obtain a higher social status in their peer group and thus hasten social maturation. Moreover, at that age, many adolescents regularly go out with peers, a social context in which most of the risk behaviors tend to occur (Van Havere et al. 2011). Therefore, involvement in risk behavior in middle adolescence might primarily express compliance with the protocol of social behavior on a night out with peers (e.g., drinking, using drugs, behaving unconventionally). We were unable to test this in our dataset. Future research is recommended to test this specific hypothesis.

Nevertheless, it is too early to conclude that those with a late onset of risk behavior (i.e., the low-high transition group in the present study) are not at risk for problems in (young) adulthood, as the risk behavior itself may lead to adverse outcomes. For example, studies have clearly demonstrated the potential harmfulness of alcohol use for the developing brain, a process which continues until early adulthood (Hiller-Sturmhöfel and Swartzwelder 2004). Tucker et al. (2005) found that youths who were not early substance users, but steadily increased their use over time, also tended to be at a relatively high risk for problems in early adulthood (Tucker et al. 2005). More recent work by Moffitt et al. demonstrated that at age 26 and 32, the adolescent limited group, in particular the males, were experiencing more mental health, physical health and economic problems than the low involvement group (Moffitt et al. 2002; Odgers et al. 2008). Thus, even though the late onset group appears to be less problematic, their behavior is not without risk, and they represent a substantial part of the adolescent population (41.8 % in our study). Therefore, they should not be overlooked with respect to preventive interventions.

### Strengths and Limitations

This study is one of the few to investigate the development of adolescent multiple risk behavior, using data from a large population based cohort study based in the Netherlands. The longitudinal design and the application of LTA allowed conclusions to be drawn with respect to transitions over time in levels of risk behavior across adolescence. Moreover, through the combined use of self-reports and parent reports, we were able to assess the predictive relationship between transition patterns and a range of childhood behavioral and family factors, which were all measured when respondents were around the age of 11, thereby limiting the risk of recall bias.

This study also has some limitations. First, the results for the risk behaviors were based on adolescent self-reporting and may be biased, most likely toward underreporting due to socially desirable answering patterns. However, when anonymity is assured, as was the case in the present study, self-report measures on risk behavior are shown to have acceptable reliability (Murray and Perry 1987). Second, data were only available until middle adolescence. It also would be interesting to study the development of risk behavior in late adolescence and young adulthood and to identify which predictors are of importance in that life phase. Third, the method we employed to study the relationship between the transition statuses and putative predictors, i.e., regression of most likely class membership on the covariates, does not take into account the different probabilities of individuals being in the same class and instead they are treated as if they all have a probability of 1.0 (Clark and Muthén 2010). However, when the entropy of classification is relatively high, as in our study (0.74), the bias due to an incorrect classification of individuals is small (Clark and Muthén 2010). Finally, our study supported the usefulness of a single risk behavior construct when studying the development of multiple risk behaviors. However, the approach is limited in that it does not reveal specific effects of risk factors on the individual risk behaviors or the possible effect that risk behaviors have on each other (Duncan et al. 1998).

### Implications

The results of our study clearly demonstrated that the development of substance use is correlated with externalizing problems, thereby underlining the need to take an integrated approach to prevention. Early onset risk behavior has been shown to be one of the strongest predictors for adverse consequences later in life and our study showed that the early onset group can be clearly identified by individual and family factors measured around the age of 11 years. Teachers can play an important role in the early identification of high risk youth, as most of the risk factors appear to be relatively easy to recognize. For example, externalizing behavioral problems around the age of 11 years appeared to be a very strong predictor, and likely to be revealed in class room situations. Finally, our results underscored the importance of timely prevention, since those involved in risk behavior at a young age appear to be unlikely to reduce their involvement over the course of adolescence.
